# Supplementary material for: Volatile Compositions of Panax ginseng and Panax quinquifolium Grown for Different Cultivation Years
Source: Foods. 2022 Dec 27;12(1):136. doi: 10.3390/foods12010136 (PMC9818133; doi:10.3390/foods12010136)

Figure S1. Discriminant analysis of four different white ginseng roots grown for 3–6 years based on volatile compound data obtained from GC–MS. (A) PCA and (B) PLS-DA score plot.

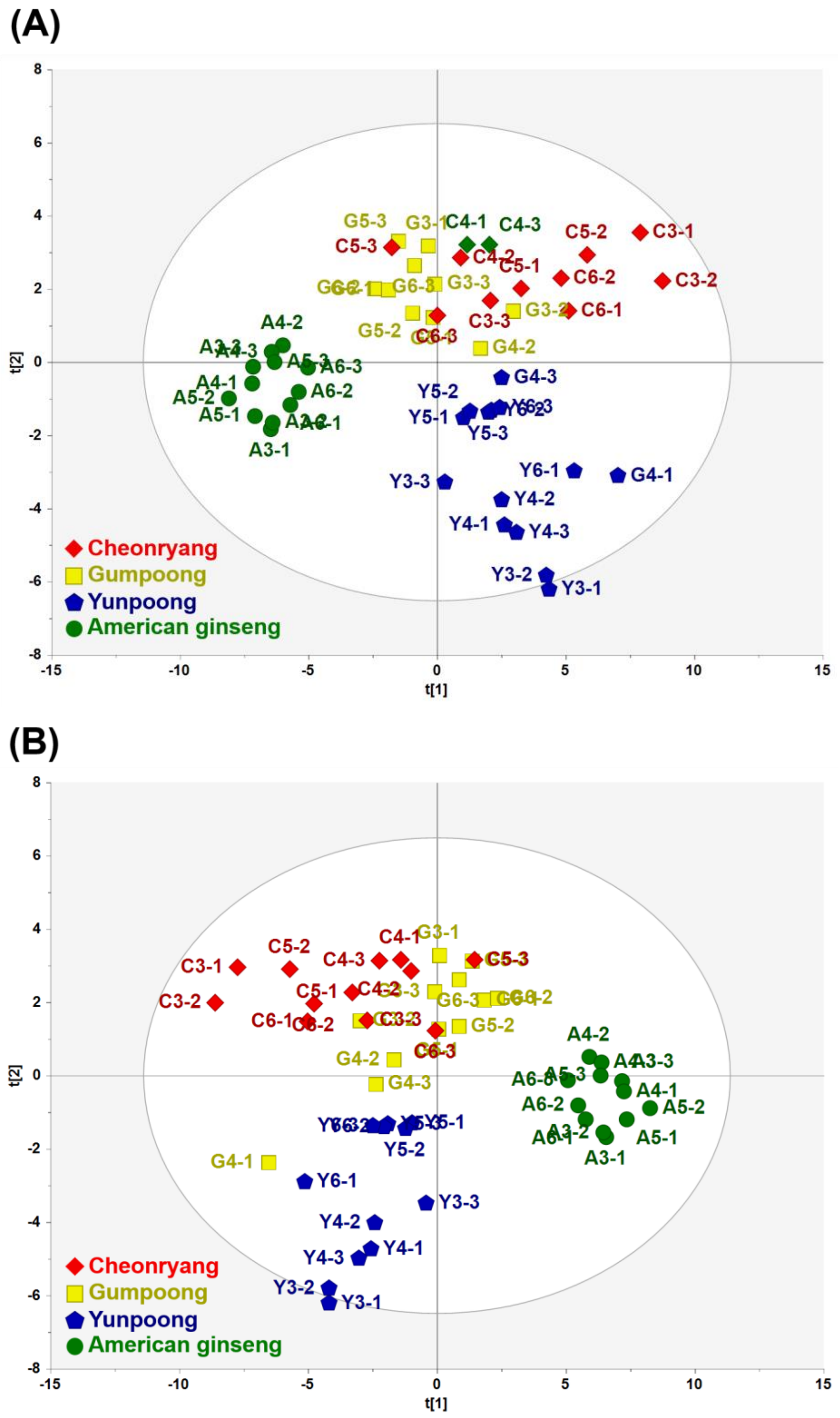

Figure S2. PLS-DA score plot of four different white ginseng roots based on volatile compound data obtained from GC–MS. (A) 3-year-old, (B) 4-year-old, (C) 5-year-old, and (D) 6-year-old ginseng roots.

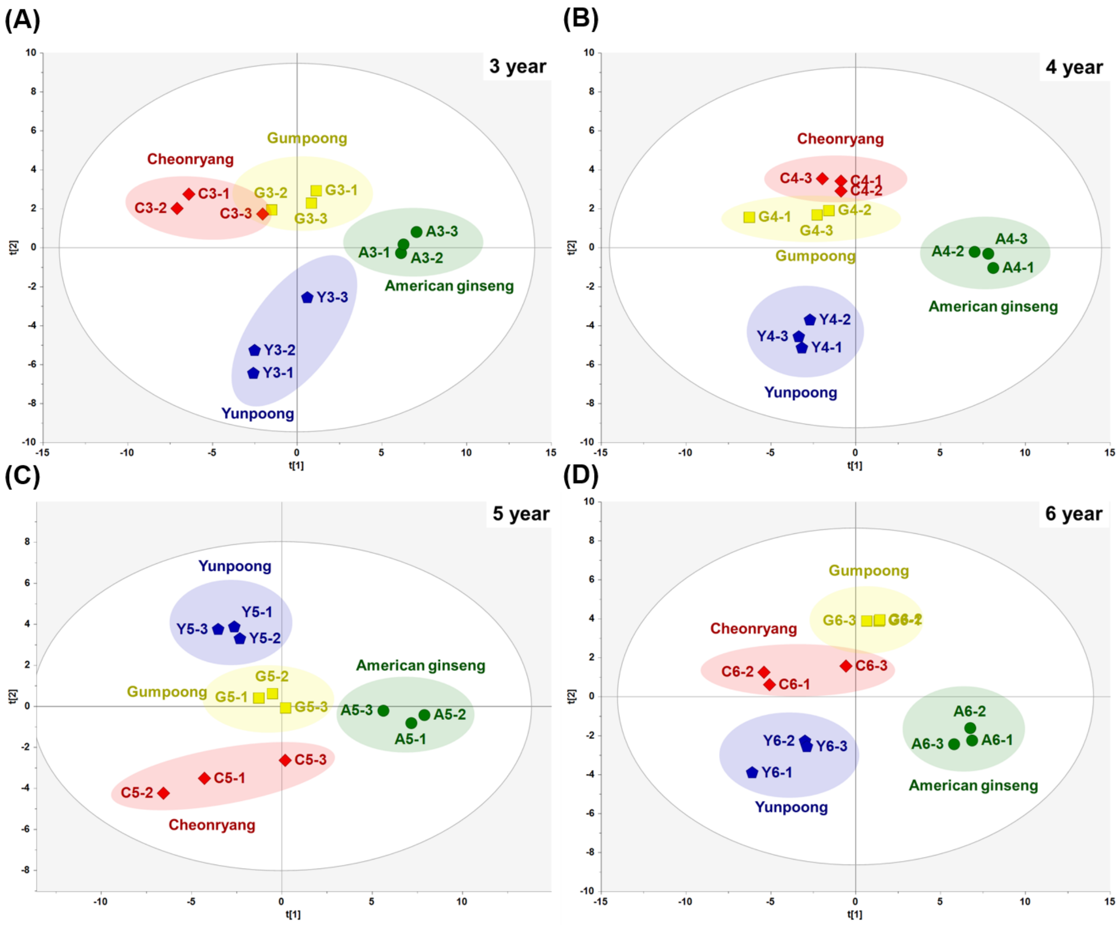

Figure S3. The VIP scores calculated from PLS-DA analysis on volatile compounds of four different white ginseng roots. (A) 3-year-old, (B) 4-year-old, (C) 5-year-old, (D) 6-year-old ginseng roots.

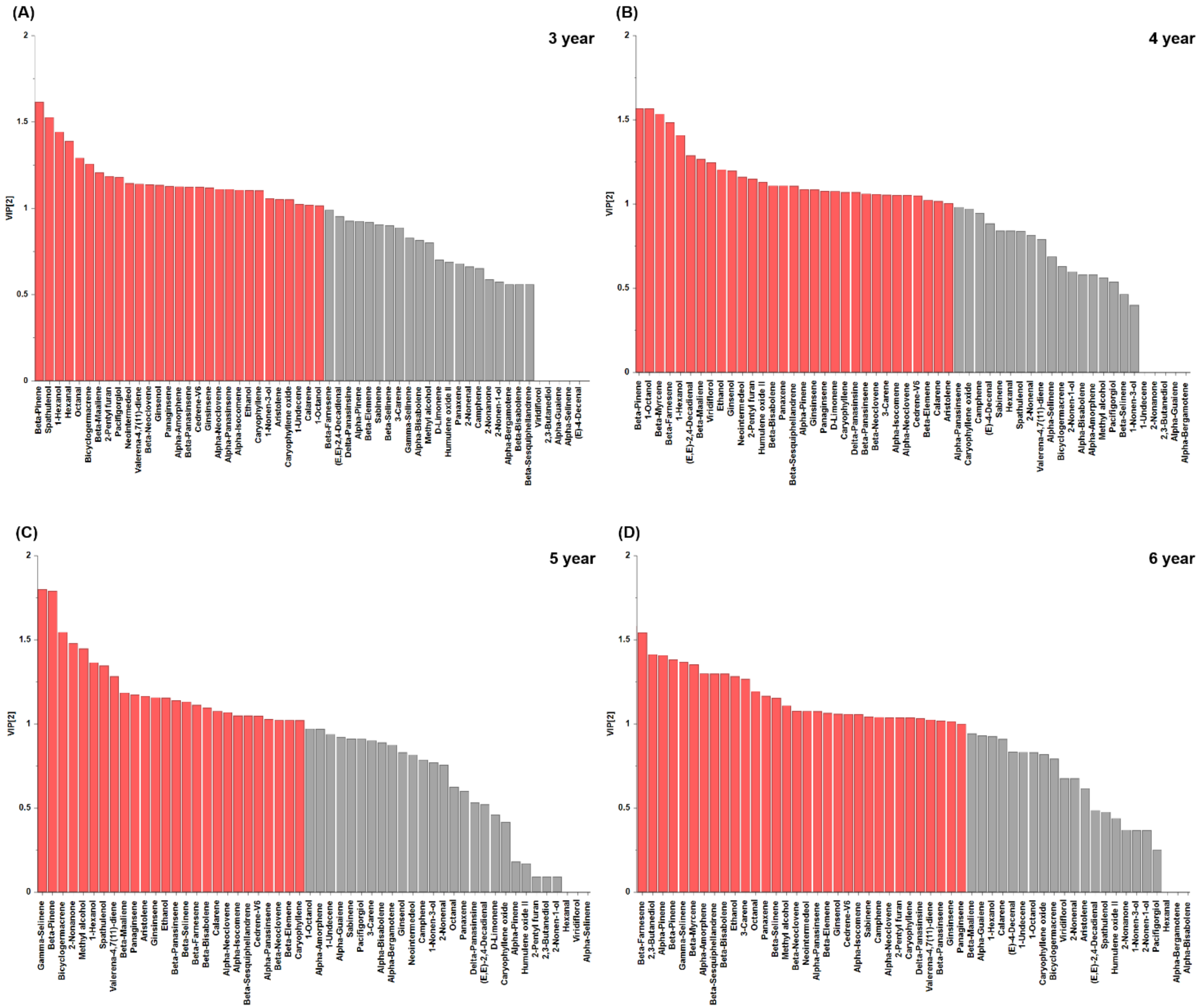

Figure S4. Heap map and HCA clustering results of the discriminant volatile compounds identified in the four different white ginseng roots grown for 3–6 years (VIP value > 1 in PLS-DA).

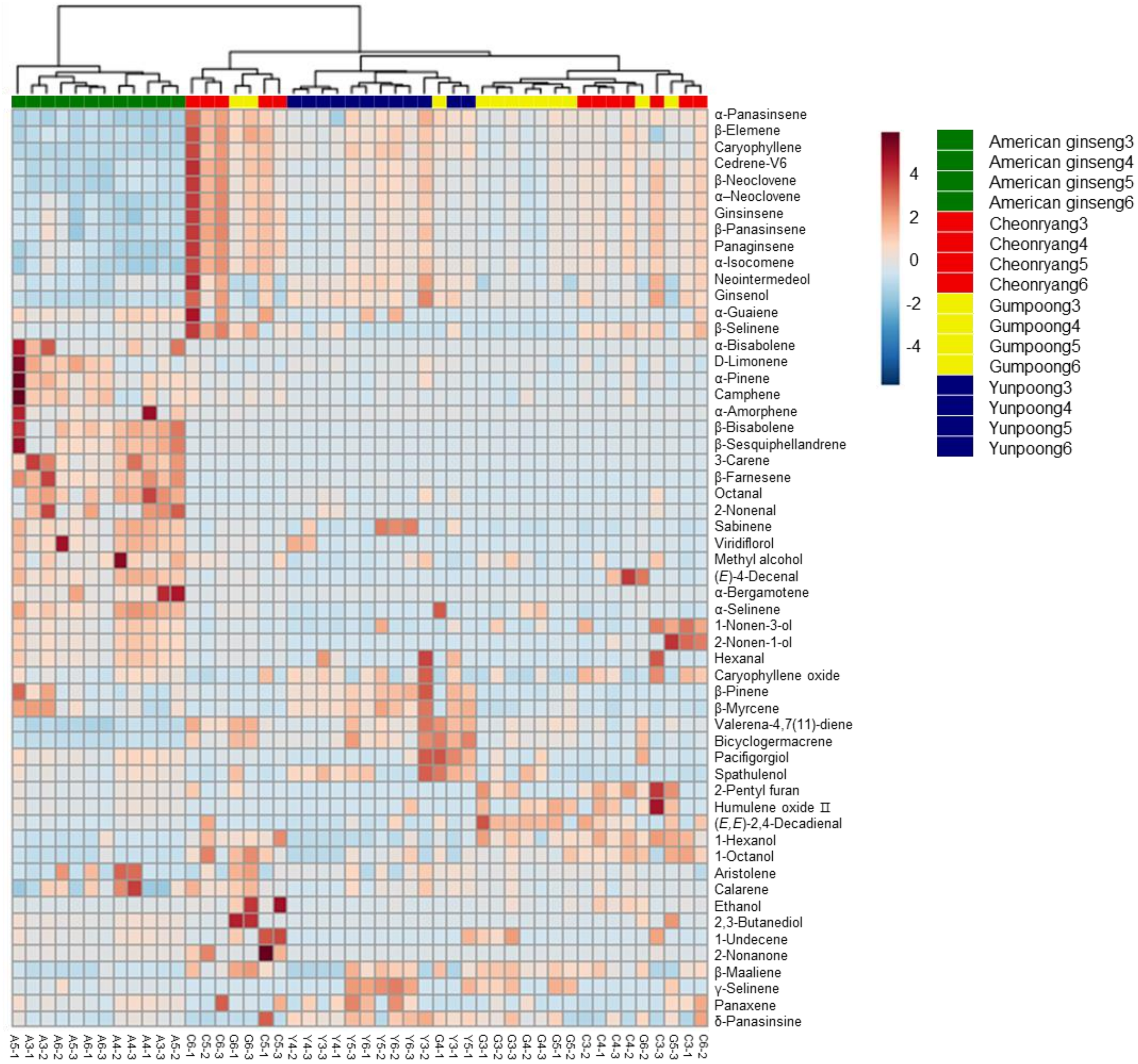

Figure S5. PLS-DA model validation of volatile compounds present in the four different white ginseng roots grown for 3–6 years by using permutation test

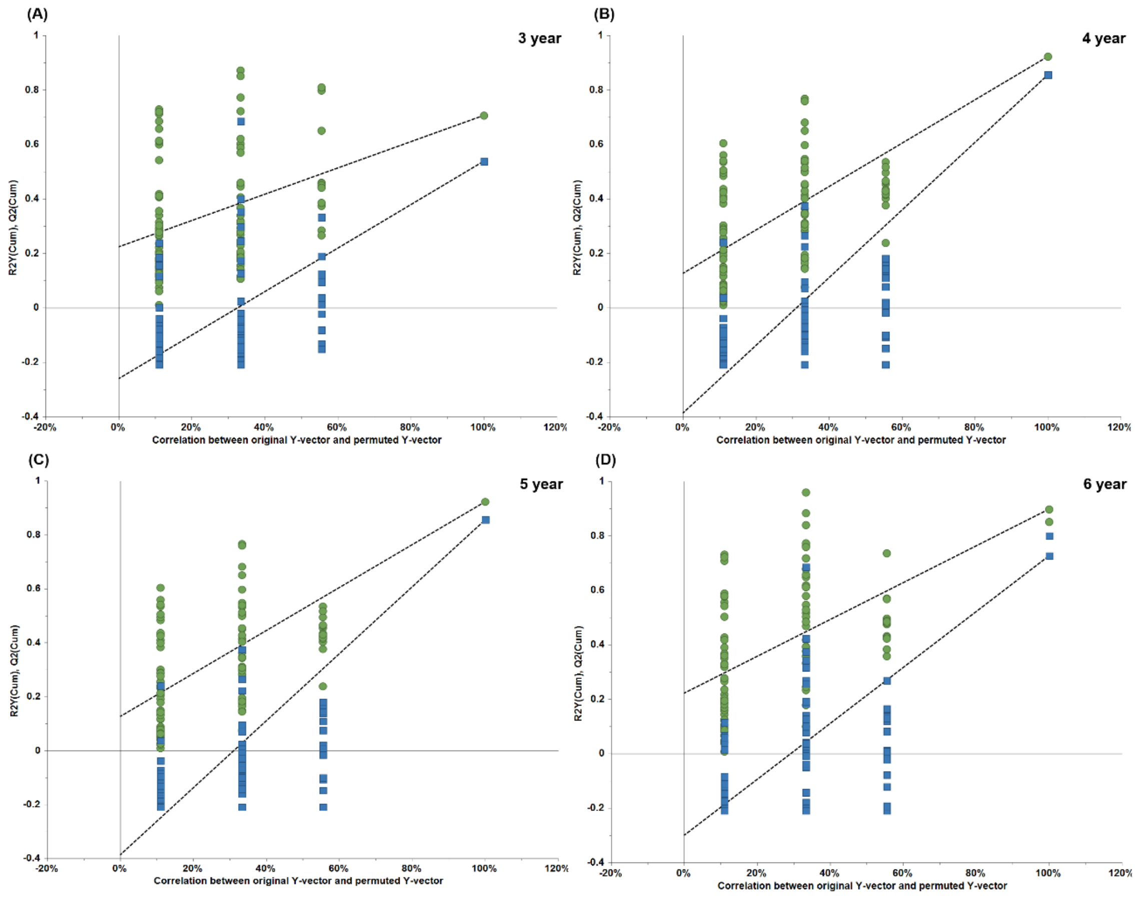

Figure S6. PCA score plot of different year white ginseng roots based on volatile compound data obtained from GC–MS. (A) American ginseng, (B)Yunpoong, (C) Gumpoong, and (D) Cheonryang.

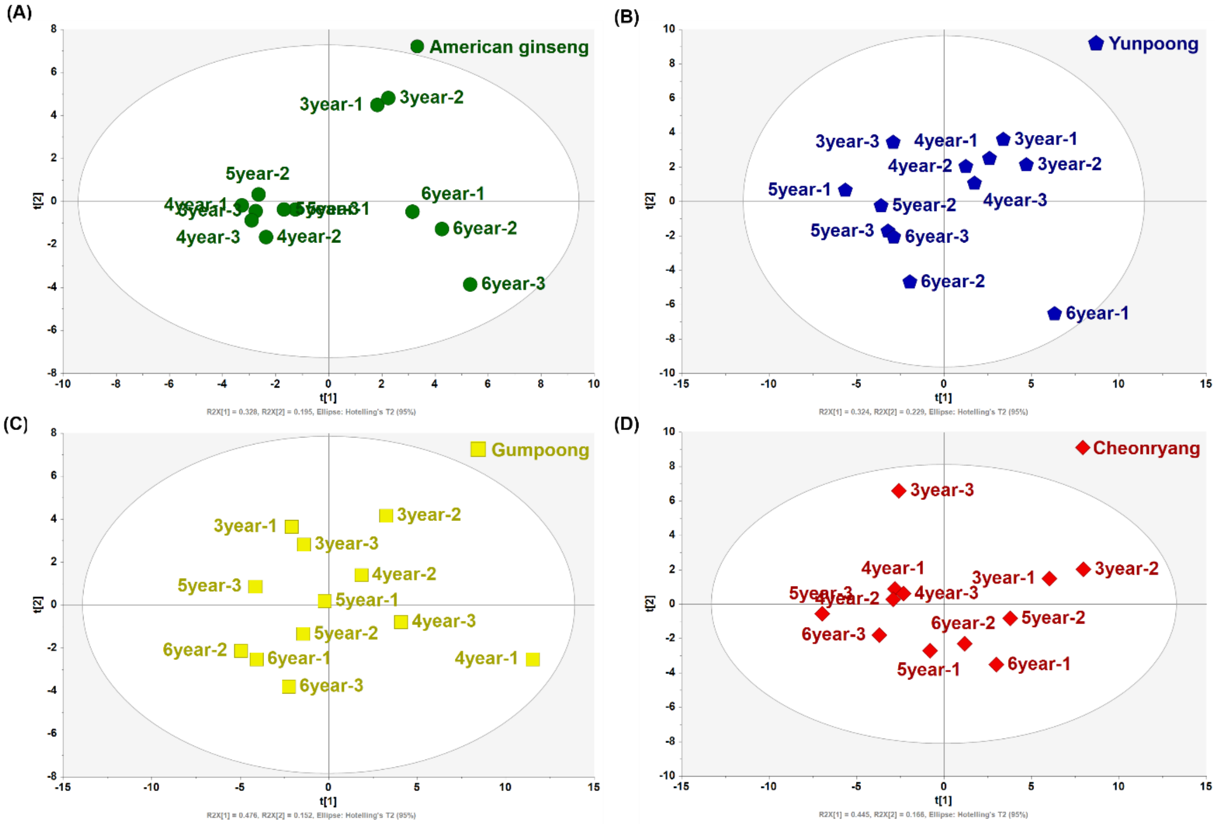

Supplement: Supplementary file 1 [file foods-12-00136-s001.zip › foods-2104009-supplementary.pdf]
